# Musculoskeletal symptoms and their impact on health-related quality of life in chronic nonbacterial osteomyelitis patients

**DOI:** 10.1186/s12969-024-00971-7

**Published:** 2024-03-06

**Authors:** Samar Tharwat, Mohammed Kamal Nassar

**Affiliations:** 1 Rheumatology & Immunology Unit, Department of Internal Medicine, Faculty of Medicine, Mansoura University, Mansoura, Egypt; 2 Department of Internal Medicine, Faculty of Medicine, Horus University, New Damietta, Egypt; 3 Mansoura Nephrology & Dialysis Unit (MNDU), Department of Internal Medicine, Faculty of Medicine, Mansoura University, Mansoura, Egypt; 4 Mansoura University Hospital, El Gomhouria St, 35511 Mansoura, Dakahlia Governorate Egypt

**Keywords:** Musculoskeletal, Pain, Health-related quality of life, Chronic nonbacterial osteomyelitis

## Abstract

**Introduction:**

Chronic non-bacterial osteomyelitis (CNO) is a rare, non-infection- related inflammatory disorder that affects children and teens. Clinical manifestations of CNO range widely from moderate, time-limited, monofocal inflammation of the bone to extreme multifocal or chronically active inflammation of the bone.

**Objectives:**

The main aim of this study was to explore the correlation between musculoskeletal (MSK) symptoms and health-related quality of life (HRQoL) in patients with CNO.

**Methods:**

Children and adults with CNO and their parents were asked to answer a web-based survey. The survey consisted of multiple questions centered around demographic, clinical and therapeutic data, MSK discomfort form based on the Nordic MSK Questionnaire and HRQoL based on Pediatric Quality of Life Inventory-4 (PedsQL-4) and PedsQL rheumatology module. The inclusion criteria included diagnosis of CNO before the age of 18. Patients who had malignancies or any chronic rheumatic, MSK, neurological disease prior to CNO onset were excluded.

**Results:**

There was a total of 68 participants, mostly females (66.2%), with median age 14 years and median disease duration 4.75 years. The median number of bones affected by CNO was 5 and ranged from 1 to 24 bones. Among the studied patients, 45 patients (66.2%) had MSK manifestations at the last month. The most commonly affected part was ankle and feet (26.5%). Regarding HRQoL, patients with MSK manifestations had lower scores than did patients without in PedsQL-4 (*p* < 0.001) including domains of physical functioning (*p* < 0.001), emotional functioning (*p* = 0.033), social functioning (*p* < 0.001) and school functioning (*p* = 0.007) in addition to lower scores in PedsQL rheumatology module (*p* < 0.001) including domains of pain and hurt (*p* < 0.001), daily activities (*p* < 0.001), treatment (*p* = 0.035), worry (*p* = 0.001) and communication (*p* < 0.001).

**Conclusion:**

MSK manifestations have a negative impact on HRQoL in CNO patients. So, early identification and treatment are highly recommended.

**Supplementary Information:**

The online version contains supplementary material available at 10.1186/s12969-024-00971-7.

## Introduction

Chronic non-bacterial osteomyelitis (CNO) is an autoinflammatory bone condition first described by Giedion et al. in 1972 as subacute and chronic “symmetrical” osteomyelitis [1]. The condition mainly affects children and teenagers, with the highest occurrence between the ages of 7 and 12. The stated rate of occurrence is between 0.4 and 10.0 per 100,000 individuals [2–4]. The incidence of CNO exhibits geographical variation, which is believed to be attributed to heightened recognition of the condition in places with higher prevalence rather than ethnic or environmental variables [5]. It is distinguished by recurrent episodes of localized bone pain manifesting as inflammation, pain, or function impairment [6]. There is a wide range of clinical symptoms, ranging from a mild, unifocal, and time-limited disease to a severe, chronically active, or recurring disease with multifocal bone lesions accompanied by cutaneous manifestations and chronic immune-mediated diseases [7]. The involvement of tissues other than bone, such as the skin, eyes, gastrointestinal tract, and lungs, has been documented [8].

CNO is characterized by diverse clinical presentations, ranging from no symptoms to intense localized pain from multiple foci [9]. It can be found in all bones, particularly in the meta- and epiphyses of long bones in children. However, it is also frequently observed in the pelvis, shoulder girdle, and spine of both children and adults [10]. According to the findings of Falip et al., out of the 31 children who were examined, 77% of them had involvement of long bones, most commonly the tibia or the femur. Involvement of the epiphysis is quite common; however, it is not always exclusive, and it frequently occurs as a component of a larger lesion that involves the metaphysis [11].

The World Health Organization (WHO) defines quality of life (QoL) as an individual’s subjective assessment of their overall well-being, taking into account their social and cultural background, personal aspirations, expectations, standards, and worries [12]. Health-Related Quality of Life (HRQoL) refers to the assessment of health status from the perspective of patients and is a valuable tool for evaluating and understanding the outcomes of diseases [13]. The significance of comprehending the influence of disease on the HRQoL of children is currently acknowledged [14]. HRQoL refers to a multifaceted notion that encompasses various dimensions such as social, emotional, and physical functioning or well-being. It is directly associated with the patient’s health condition [15].

Nevertheless, only a small number of studies have examined the musculoskeletal (MSK) symptoms in patients with CNO and how they affect their HRQoL. So, the aim of this study was to evaluate MSK discomfort and its correlation with HRQoL in CNO patients.

## Patients and methods

### Study design and setting

This cross-sectional study was carried out on 68 CNO patients from different countries. It was a study based on surveys, and either the participants themselves or their parents were needed to fill out an online questionnaire that was self-administered and created using Google Forms. The study was open to all CNO patients from various nations who were diagnosed with the condition before turning 18 years old. Patients who had been diagnosed with malignancies or any chronic rheumatic, MSK, or neurological disease prior to the onset of CNO were not allowed to participate in the study from the very beginning. The questionnaire was distributed to all potential participants at random via social media platforms (such as Facebook and WhatsApp) between the 6th of January and the 28th of May 2020. The questionnaire is available at the following link: https://docs.google.com/forms/d/e/1FAIpQLSeOfTFftHGm46sWjRljDAbdWXgLcLxd80sGY1EheJhfFYTH0w/viewform. After that, they were taken to a website that detailed the aim of the study and provided them with directions on how to complete the questionnaire. Participants were assured of their anonymity as well as the confidentiality of their data. Everyone who agreed to participate in the study was directed to the Google Form. It was understood that providing responses to all of the questions and thereafter submitting them counted as consent to take part in the study.

### Ethical consideration

This research was conducted in compliance with the principles of the Helsinki Declaration [16], and the study protocol was approved by the Institutional Research Board of the Faculty of Medicine at Mansoura University (approval registration number: R.23.12.2436).

### Sociodemographic, clinical, and therapeutic data

Moreover, all participants were asked to complete additional questions concerning the duration of the disease, the number of bones that were impacted by CNO, and any other associated comorbidities that manifested after the onset of CNO.

### CNO therapeutic data

Therapeutic data were also obtained from the participants. They were questioned about the medications they used to control their CNO illness, including corticosteroids, bisphosphonates, conventional disease-modifying antirheumatic drugs (cDMARDs), biological DMARDs (bDMARDs), nonsteroidal anti-inflammatory drugs (NSAIDs), pain medications, antibiotics, physical therapy, surgery, and radiation.

### The nordic musculoskeletal questionnaire

The primary purpose of the Nordic Musculoskeletal Questionnaire (NMQ-E) was to investigate the presence and distribution of MSK symptoms in a comprehensive manner across a variety of body regions. The participant was asked to determine the approximate position of the parts of his/her body that were bothering him/her [17].

### The pediatric quality of life inventory (PedsQL-4)

The pediatric quality of life inventory (PedsQL-4) Measurement Model [18] is a modular approach to assessing HRQoL in both healthy children and adolescents, as well as those with acute and chronic health problems. The PedsQL-4 allows for the evaluation of both global (generic) and disease-specific (disease-specific) quality of life. Physical functioning (8 items), emotional functioning (5 items), social functioning (5 items), and school functioning (5 items) comprise the four scales comprising this generic battery. Three standardized summary scores can be derived from these four main scales: a Total Quality of Life Score, a Physical Health Summary Score (based on the physical functioning items), and a Psychosocial Health Summary Score (combining emotional, social, and school items). The respondent is asked to identify how much of a difficulty each item has been in the past month on each of the PedsQL-4 scales, with the response alternatives of 0 = never, 1 = almost never, 2 = sometimes, 3 = often, and 4 = very always. To produce scale and summary scores, the item scores are first reverse coded, then linearly translated to a scale with 100 points, and then averaged. Greater scale and summary scores indicate a greater overall quality of life.

### The PedsQL rheumatology module

The 22-item multidimensional PedsQL 3.0 Rheumatology Module Scales include the following categories: (1) Pain and Hurt (with four items), (2) Daily Activities (with five items), (3) Treatment (with seven items), Worry (with three items), and (4) Communication (with three items). Higher scores indicate a better HRQOL (fewer difficulties or symptoms), and the format, instructions, and Likert scale as well as the manner of scoring are all the same as those found in the PedsQL-4 Generic Core Scales [19].

### Statistical analysis

To do analysis on the collected data, the Statistical Package for Social Science (SPSS) version 22 program was utilized. When presenting quantitative data, we used mean and standard deviations (SD) for parametric variables and median (min-max) for nonparametric variables. When presenting qualitative data, we used percentages and numbers. The Shapiro–Wilk test was carried out to ascertain whether the distribution of the variable was normal. The independent samples t test was used to determine whether there was a statistically significant difference between two groups when the data were normally distributed; however, the Mann-Whitney test was utilized when the variables in question were not parametric. To do comparisons across qualitative variables, we either used the Chi-square test or the Fisher exact test, as appropriate. A *p* value of less than 0.05 was considered statistically significant.

## Results

During the course of the study, there were 92 people who clicked on weblinks that sent them to the online survey, but only 80 of those people actually filled it out. Twelve of them were disqualified for various reasons, including the fact that three of them had an associated malignancy, six of them lacked clinical data, two of them were identified as having systemic lupus erythematosus, and one of them had Marfan syndrome before the development of CNO disease. Participants were assigned to two groups according to the presence of MSK manifestations in the last month.

**Fig. 1:**
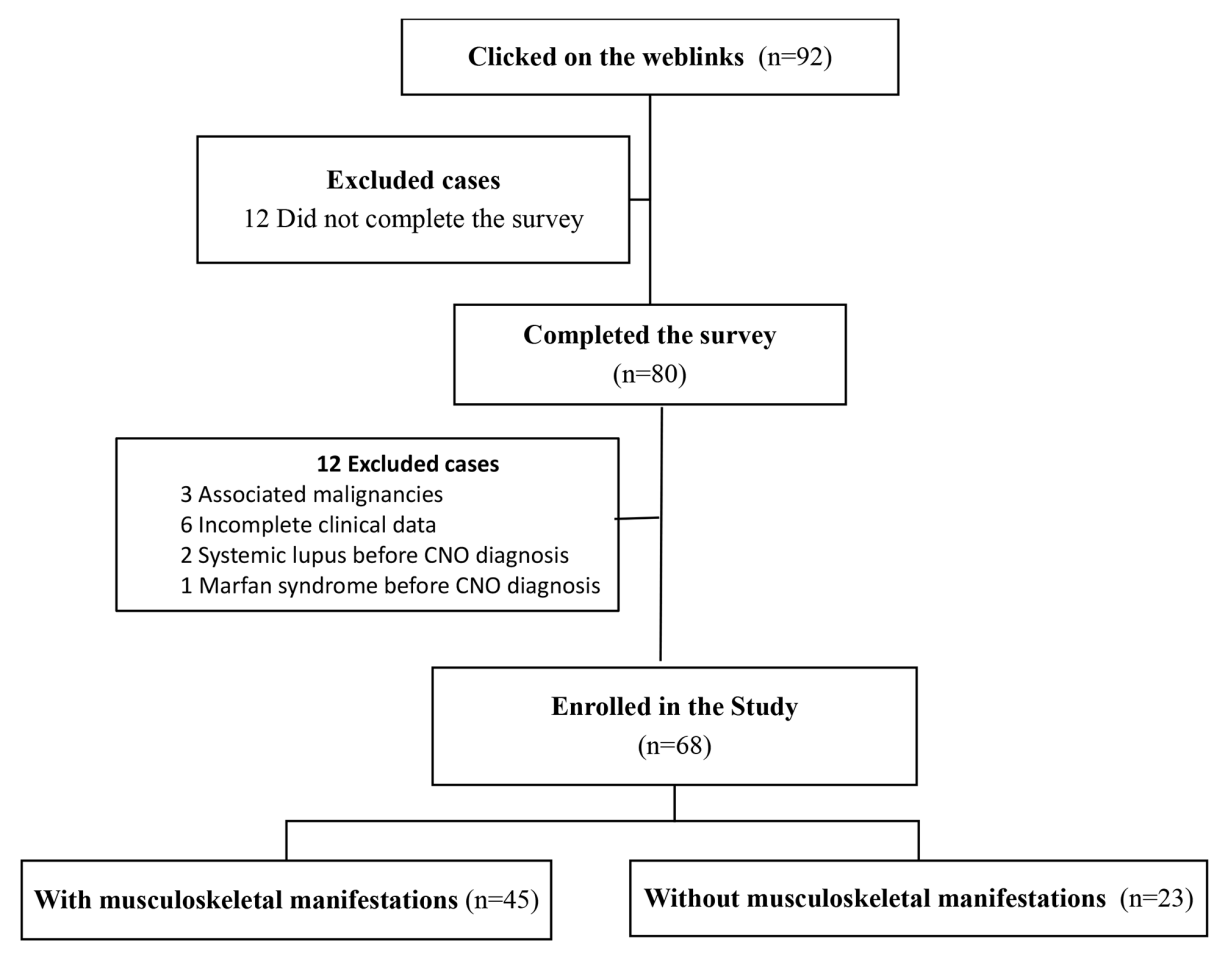
The flowchart of the study

The study included 68 CNO participants—45 with MSK manifestations and 23 without. The flowchart of the study is illustrated in Fig. 1.The median age was 14 years; most of them (86.8%) were below the age of 20 years, and about two-thirds (66.2%) were females. The median disease duration was 4.75 years, and the median number of bones affected by CNO was 5. About one-half (54.4%) had associated comorbidities; the most associated was psoriasis (17.6%), followed by juvenile idiopathic arthritis (8.8%). Other coexisting medical conditions included bronchial asthma (4 patients), celiac disease (4 patients), Von Willebrand disease (2 patients), and hypertension (2 patients). There was no statistically significant difference between those with MSK manifestations and those without, regarding age, disease duration and associated JIA. However, MSK manifestations were more prevalent in females (*p* = 0.005). Also, psoriasis was statistically significantly present in those with MSK manifestations (*p* = 0.048). Regarding treatment used, NSAIDs were the most commonly administered drugs (76.5%), followed by physical therapy (41.2%), methotrexate (MTX) (41.2%), and bisphosphonates (35.3%). Patients with MSK manifestations significantly used bisphosphonates (*p* = 0.006) and pain medications (*p* = 0.005). Other clinical and therapeutic data are illustrated in Table 1.

**Table 1:** Baseline Characteristics according to the presence or absence of MSK symptoms in the last month in the studied CNO patients (*n* = 68)

Variable *n* (%), median (min-max), mean ± SD	Study sample (*n* = 68)	Musculoskeletal manifestations		*p* value
		With (*n* = 45)	Without (*n* = 23)	
Age, years < 20 20–40 > 40	14 (5–62) 59 (86.8) 6 (8.8) 3 (4.4)	15 (5–62) 36 (80) 6 (13.3) 3 (6.7)	13 (8–19) 23 (100) 0 0	0.151 0.083
Gender				
Male Female	23 (33.8) 45 (66.2)	10 (22.2) 35 (77.8)	13 (56.5) 10 (43.5)	**0.005** ^*^
The survey completed by				
The patient The parent	13 (19.1) 55 (80.9)	11 (24.4) 34 (75.6)	2 (8.7) 21 (91.3)	0.192
Country				
USA UK Others	35 (51.5) 14 (20.6) 19 (27.9)	21 (46.7) 11 (24.4) 13 (28.9)	14 (60.9) 3 (13) 6 (26.1)	0.449
Disease duration, years < 5, n (%) ≥ 5, n (%)	4.75 (0.17-24) 34 (50) 34 (50)	5 (0.17-24) 21 (46.7) 24 (53.3)	4 (0.33-13) 13 (56.5) 10 (43.5)	0.155 0.609
Number of bones affected by CNO < 10, n (%) ≥ 10, n (%)	5 (1–24) 59 (86.8) 9 (13.2)	5 (1–24) 37 (82.2) 8 (17.8)	4 (1–18) 22 (95.7) 1 (4.3)	0.282 0.153
Associated comorbid conditions Psoriasis Juvenile idiopathic arthritis Inflammatory bowel disease Others	37 (54.4) 12 (17.6) 6 (8.8) 6 (8.8) 12 (17.6)	28 (62.2) 11 (24.4) 5 (11.1) 6 (13.3) 8 (17.8)	9 (39.1) 1 (4.3) 1 (4.3) 0 4 (17.4)	0.070 0.048* 0.565 0.089 1
Treatments used for CNO disease				
Corticosteroids Bisphosphonates	21 (30.9) 24 (35.3)	17 (37.8) 21 (46.7)	4 (17.4) 3 (13)	0.085 0.006^*^
cDMARDs				
Sulfasalazine Methotrexate	8 (11.8) 28 (41.2)	5 (11.1) 22 (48.9)	3 (13) 6 (26.1)	0.815 0.071
bDMARDs				
Adalimumab Etanercept Infliximab Golimumab Certolizumab pegol Abatacept Tocilizumab	9 (13.2) 7 (10.3) 11 (16.2) 3 (4.4) 1 (1.5) 1 (1.5) 2 (2.9)	6 (13.3) 6 (13.3) 10 (22.2) 3 (6.7) 1 (2.2) 1 (2.2) 2 (4.4)	3 (13) 1 (4.3) 1 (4.3) 0 (0) 0 (0) 0 (0) 0 (0)	0.973 0.249 0.058 0.205 0.471 0.471 0.305
**Others**				
NSAIDs Pain medications Antibiotics Physical therapy Surgery Radiation	52 (76.5) 21 (30.9) 5 (7.4) 28 (41.2) 9 (13.2) 1 (1.5)	34 (75.6) 19 (42.2) 5 (11.1) 20 (44.4) 7 (15.6) 1 (2.2)	18 (78.3) 2 (8.7) 0 (0) 8 (34.8) 2 (8.7) 0 (0)	0.804 0.005^*^ 0.097 0.444 0.43 0.471

*p < 0.05

Table 2 shows the total number of bDMARDs administered by the participants. About two-thirds (63.2%) did not administer any of the bDMARDs, while 27.9% administered at least 1 agent, 5.9% administered at least 2 agents, and only one patient received 3 agents and another one received 4 agents. There was no statistically significant difference between those with MSK manifestations and those without regarding the number of bDMARDs that were received previously (*p* = 0.133).

**Table 2:** Biological agents used for management of the study CNO patients (*n* = 68)

Variable *n* (%)	Study sample (*n* = 68)	Musculoskeletal manifestations		*p* value
		With (*n* = 45)	Without (*n* = 23)	
No drug At least 1 drug At least 2 drugs At least 3 drugs At least 4 drugs	43 (63.2) 19 (27.9) 4 (5.9) 1 (1.5) 1 (1.5)	24 (53.3) 16 (35.6) 3 (6.7) 1 (2.2) 1 (2.2)	19 (82.6) 3 (13) 1 (4.3) 0 0	0.133

**Fig. 2:**
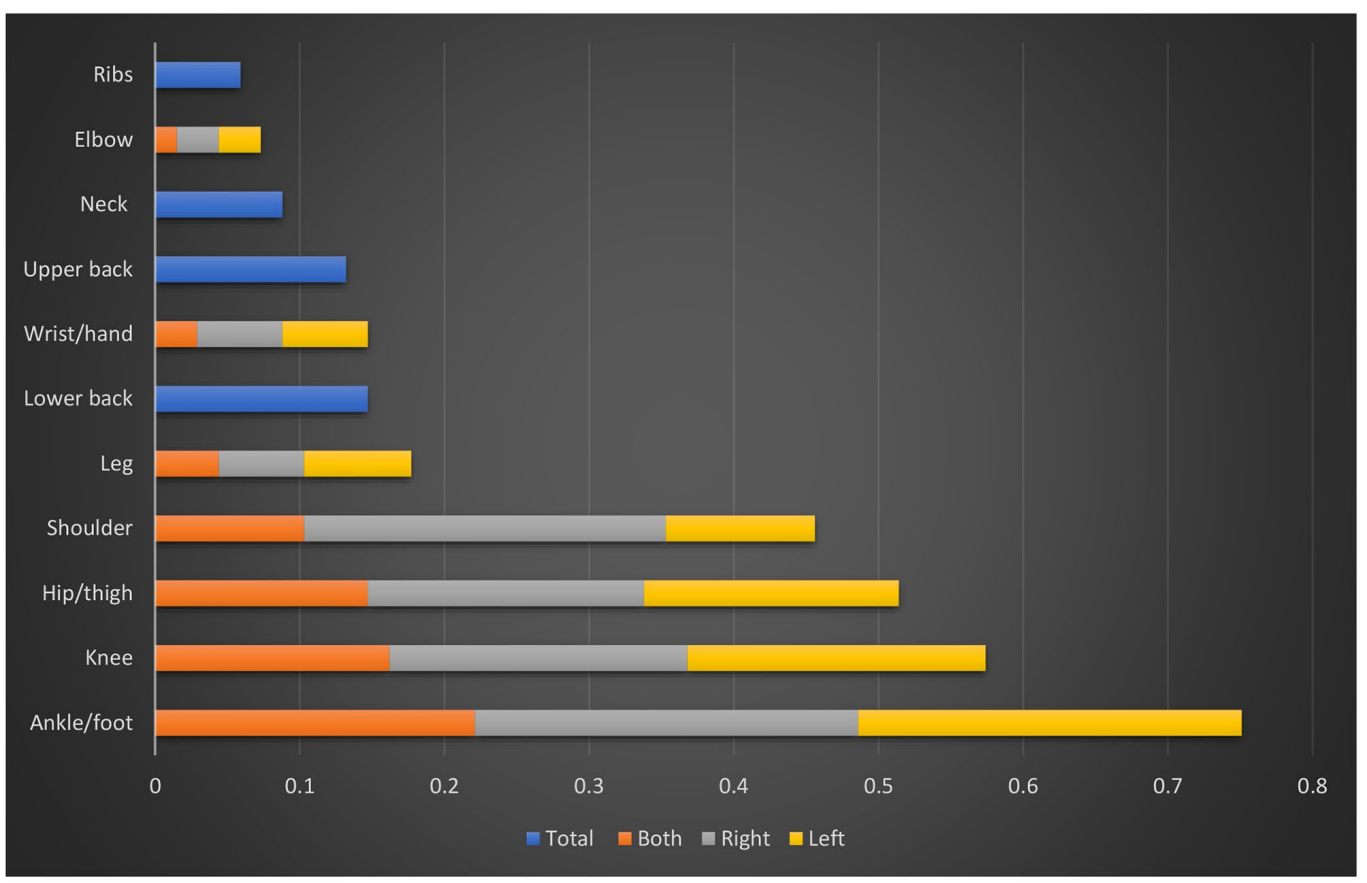
Distribution of musculoskeletal manifestations (ache, pain, discomfort, numbness) in the last month among the studied CNO patients (*n* = 68)

The frequency and distribution of MSK symptoms (ache, pain, discomfort, and numbness) in the previous-month period are shown in Fig. 2. According to the body regions, the maximum number of patients encountered pain in the last month in the right and left ankle/foot (26.5%) and right shoulder (25%), followed by the right and left knee (20.6%), while the neck (8.8%) and ribs (5.9%) were the least affected body areas.

**Fig. 3:**
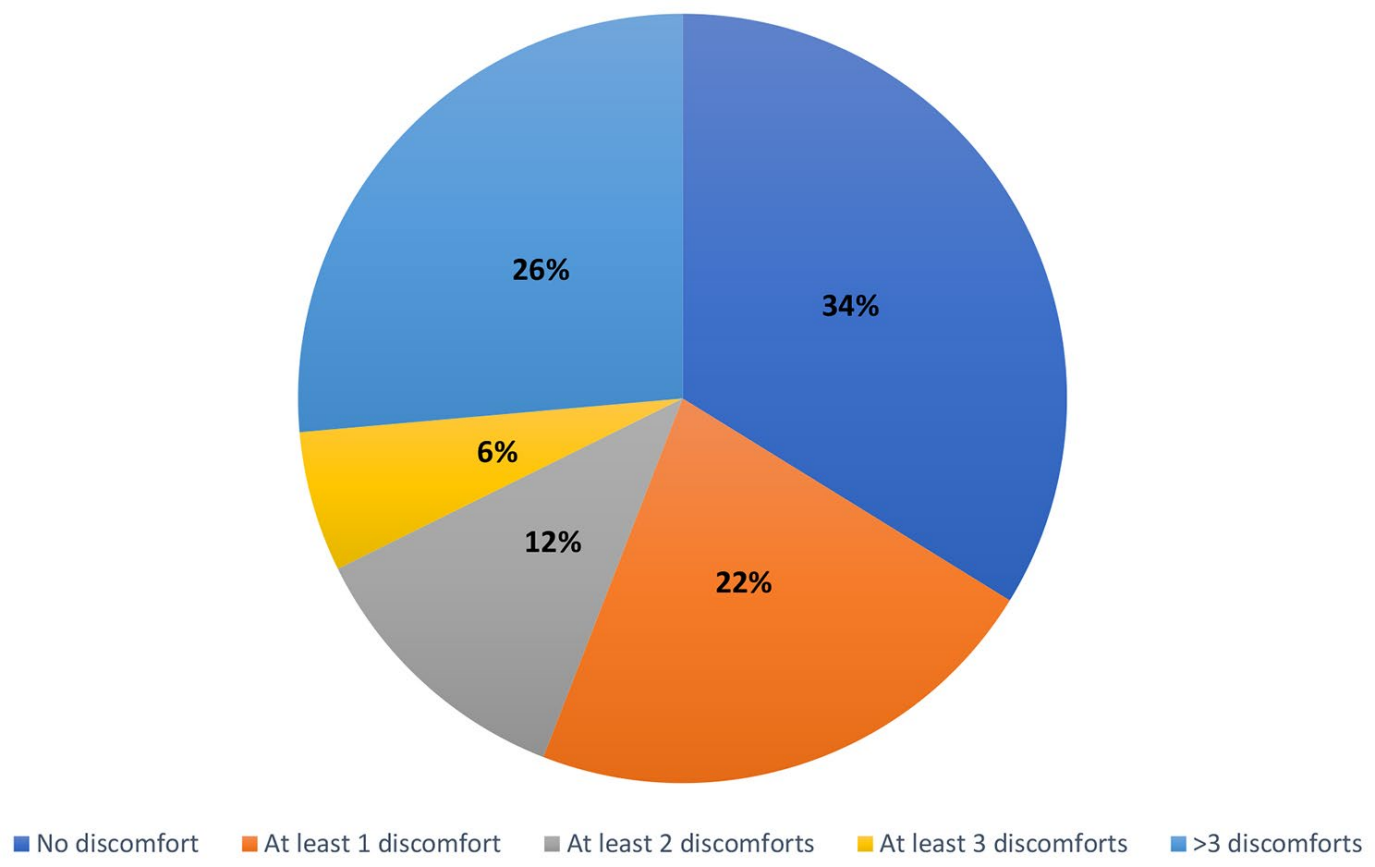
Number of MSK discomforts per individual in the last month among the studied group (*n* = 68)

According to the Nordic MSK questionnaire, most of the studied patients (66%) reported MSK symptoms in the last month; 22% described at least 1 site of discomfort; 12% had at least 2; 6% had at least 3; and 26% had > 3 body sites of MSK discomfort, as illustrated in Fig. 3.

The study employed the score of PedsQL-4.0 generic core scale domains according to the presence or absence of MSK symptoms. Patients with MSK symptoms had statistically significantly lower scores than did patients without in all domains. Additionally, when we employed the PedsQL3.0 rheumatology module, we found that those with MSK symptoms had statistically significant lower scores in all domains, as shown in Table 3.

**Table 3:** Results of questionnaires; PedsQL4.0, PedsQL 3.0 rheumatology module according to the presence or absence of MSK symptoms in the last month in the studied CNO patients (*n* = 68)

Variables median (min-max)	CNO patients (*n* = 68)	Musculoskeletal manifestations median (min-max)		*p* value
		With (*n* = 45)	Without (*n* = 23)	
**PedsQL4.0 generic core scale**				
Physical functioning	48.4 (0-96.88)	40.62 (0–75)	65.63 (15.63–96.88)	**< 0.001** ^*^
Emotional functioning	55 (0-100)	50 (0–90)	60 (10–100)	**0.033** ^*^
Social functioning	75 (25–100)	70 (25–100)	85 (50–100)	**< 0.001** ^*^
School functioning	50 (0–95)	45 (0–80)	60 (25–95)	**0.007** ^*^
Physical Health Summary Score	48.4 (0-96.88)	40.63 (0–75)	65.63 (15.63–96.88)	**< 0.001** ^*^
Psychosocial Health Summary Score	60 (10-98.33)	55 (10-81.67)	68.33 (35-98.33)	**0.001** ^*^
Total scale score	56.56 (7.50-97.97)	50.94 (7.50–80)	65.63 (31.72–97.97)	**< 0.001** ^*^
**PedsQL3.0 rheumatology module**				
Total scale score	52.25 (8.81–96.25)	48.38 (8.81–71.81)	61.79 (33.81–96.25)	**< 0.001** ^*^
Pain and hurt	40.63 (0-87.50)	31.25 (0-56.25)	50 (0-87.50)	**< 0.001** ^*^
Daily activities	87.50 (0-100)	75 (0-100)	95 (55–100)	**< 0.001** ^*^
Treatment	55.36 (7.14–100)	53.57 (7.14–85.71)	57.14 (35.71–100)	**0.035** ^*^
Worry	41.67 (0-100)	41.67 (0–75)	50 (8.33–100)	**0.001** ^*^
Communication	41.67 (0-100	33.33 (0-83.33)	66.67 (25–100)	**< 0.001** ^*^

^*^p < 0.05

## Discussion

To the best of our knowledge, this is the first detailed study encountered by CNO patients that utilizes a Nordic MSK questionnaire to evaluate MSK symptoms among these patients. The study includes those diagnosed with CNO and provides data on MSK manifestations and their relation to HRQoL in CNO patients. The present study revealed that CNO patients had a heavy MSK symptom burden, which was associated with worse scores of HRQoL either in their physical, psychological, or rheumatological aspects.

CNO seems to be the standard term for long-lasting bone inflammation, no matter where it shows up (single or multiple sites) or how it progresses (self-limiting, persistently active vs. recurring) [20]. Previously considered a mild and self-limiting condition, CNO is now considered a severe and, in some instances, fatal condition due to chronic and severe pain as well as possible complications, including vertebral body fractures [21]. Despite the fact that the incidence of CNO has been reported to be as low as 4 cases per million children [22], various centers have witnessed an increase in the number of cases as awareness has been raised [23, 24].

In this study, the median age of the included CNO patients was 14 years; most of them (86.8%) were below the age of 20 years, and about two-thirds (66.2%) were females. In general, it appears that girls and young women are afflicted more commonly than boys and men (about 2:1) [25], and the majority of patients show symptoms between the ages of 7 and 9 years old [21]. CNO is infrequently observed in adults but can manifest in individuals of all age groups [9]. Females are more likely to be affected than males, and the progression of the disease varies greatly from case to case [26, 27]. Within European cohorts, there is a higher likelihood of females being affected [6, 28, 29]. while a male preponderance is reported in the Latin American and Indian series [30, 31].Obtaining a diagnosis for this condition often requires a year, on average, due to its uncommonness. However, 7% of patients experience a delay of over 5 years before receiving a diagnosis [32].

In our cohort, most of the CNO participants had MSK manifestations in the last month before the study. Pain is the most prevalent and prominent symptom of CNO [33]. It is typical for complaints to wax and wane, and an acute onset of pain may occur, necessitating a rapid diagnostic workup and prompt treatment [29]. Nocturnal exacerbations of discomfort and potential disruption of sleep are commonly observed, accompanied by localized tenderness at the affected area in the child [21].

When we questioned our cohort about additionalcomorbidities, we discovered that about one-half (54.4%) had associated comorbidities; the most common one was psoriasis (17.6%), followed by juvenile idiopathic arthritis (8.8%). When any autoimmune or autoinflammatory condition was taken into consideration, 50% of children diagnosed with CNO had at least one inflammatory comorbidity [34]. Pustular skin disease is a significant aspect or coexisting condition of CNO [29]. The prevalence of pustulosis/acne-like skin illness remains constant over time, whereas the incidence of inflammatory bowel disease (IBD) increases with the course of the disease [35]. According to the findings of other studies that investigated the connection between CNO and various auto-inflammatory diseases, there was a correlation between CNO and palmoplantar pustulosis, IBD, and psoriasis, as well as relatives who had chronic arthritis [22, 23, 36].

In this study, MSK manifestations were more prevalent in females (*p* = 0.005). Also, psoriasis was statistically significantly present in those with MSK manifestations (*p* = 0.048). In this regard, Girschick et al. described a cohort of 486 patients from around the world, which revealed that females (64%) are predominantly afflicted by CNO [29]. CNO patients with severe and multiple major bone lesions have been found to have a higher occurrence of concurrent arthritis or psoriasis [34, 37]. Also, recent data suggest that around 50% of individuals with CNO exhibit additional involvement outside of the bones, primarily in the form of psoriasis and palmoplantar pustulosis, IBD, and arthritis. However, the prevalence of specific co-morbidities varies considerably among the cohorts that have been studied [29, 34, 36, 38].

Regarding treatment used for our CNO cohort, NSAIDs were the most administered drugs (76.5%), followed by physical therapy (41.2%), MTX (41.2%), and bisphosphonates (35.3%). While NSAIDs are often recommended, particularly for children, their effectiveness is generally considered to be modest [30]. According to the current understanding of the pathophysiology of CNO, NSAIDs can potentially address the excessive activity of osteoclasts by reducing the generation of prostaglandins through the inhibition of cyclooxygenase enzymes. Prostaglandins play a crucial role in the process of osteoclast differentiation and activation. Moreover, NSAIDs influence the processing of pain, which accounts for their prompt analgesic effects [2].

In this study, patients with MSK manifestations significantly used bisphosphonates (*p* = 0.006) and pain medications (*p* = 0.005). Bisphosphonates have emerged as a highly efficacious second-line therapy for CNO subsequent to the failure of NSAIDs [39]. As second-line treatments for CNO, tumor necrosis factor alpha (TNF-α) inhibitors and the bisphosphonate pamidronate are frequently prescribed in Europe [40]. A multicenter study conducted by Bhat et al. found that bisphosphonate treatment was effective in 68.5% of all cases involving patients [6]. Although bisphosphonates do affect bone remodeling, they do not cause damage to developing skeletons or impede normal growth [41]. Various treatment modalities have been deliberated for patients who do not exhibit a favorable response to NSAIDs or who have predominant vertebral involvement. These alternatives consist of MTX, sulfasalazine (SSZ), biopharmaceutical drugs, specifically TNF-α inhibitors, and bisphosphonates [40].

In this study, about two-thirds (63.2%) did not administer any of the bDMARDs, while 27.9% administered at least 1 agent, 5.9% administered at least 2 agents, and only one patient received 3 agents and another one received 4 agents. Notwithstanding the growing recognition of CNO over the last few decades, the lack of evidence-based treatments continues to pose a challenge to clinical management [42]. Currently, there are no approved therapies available for the treatment of CNO. NSAIDs, MTX, sulfasalazine, pamidronate (PAM), anti-IL1 agents, and TNFα inhibitors have all been shown to work in many studies [29, 34].

According to the body regions, the maximum number of patients encountered pain in the last month in the right and left ankle/foot (26.5%) and right shoulder (25%), while the neck (8.8%) and ribs (5.9%) were the least affected body areas. The distribution of lesions has been exhaustively studied, typically utilizing whole body MRI at presentation and throughout the course of the disease, and typically affects the metaphyses of long bones, particularly in the lower extremities close to the knees and ankles [43].CNO can be found in all bones, but it is more frequently seen in the metaphyses and epiphyses of the long bones in children. However, it is not unusual to find CNO in the pelvis, shoulder girdle, and spine of both children and adults. The presentation of CNO in the jaw is one that is frequently overlooked [33, 44, 45].

According to the Nordic MSK questionnaire, most of the studied patients (66%) reported MSK symptoms in the last month; 22% described at least 1 site of discomfort;12% had at least 2; while 6% had at least 3; and 26% had > 3 body sites of MSK discomfort. Similar to other systemic inflammatory diseases, increased MSK pain can be a substantial comorbidity or consequence with a notable psychosocial impact [46].

The concept of HRQoL has gained significant recognition in recent years as an outcome that is useful to patients, as it reflects the effects of chronic illness on different health conditions [47]. In the present study, CNO patients with MSK symptoms had statistically significantly lower scores than did patients without in all domains of HRQoL. Although the treatment is advanced, patients with CNO still experience poor HRQoL [48]. In a study that comprised 18 CNO patients from a single center in Turkey, the authors discovered that the HRQoL of CNO patients was negatively affected, even though the treatment was effective [48]. These adverse consequences of CNO were detected not only in the patients’ physical activities but also in their social lives, emotional lives, and academic lives as well [48]. Another study demonstrated comparable deterioration in the physical and academic performance of patients with CNO, as assessed through the utilization of PedsQL-4. Similarly, the aforementioned study documented the presence of anxiety and difficulties in communication among individuals with CNO [49].

There are some limitations to this study that need to be addressed. One limitation of this study is that no matter who filled out the questionnaires, the patient and parent-reported measurements were analyzed together. This was done regardless of who completed the questionnaires. Even though this combination maximizes the total sample size, there may be differences in perceptions between children and proxies, particularly for children who are older. It is necessary to evaluate our HRQoL findings with respect to the treatments that were administered to the children who were included in this cohort. Children who are treated in a significantly different manner may experience varying patterns of HRQoL. We included six patients with JIA who were diagnosed after the onset of CNO in the study, which could potentially have a substantial impact on the study’s results. Due to the fact that CNO is an uncommon disease, we were compelled to do so, as it was somewhat challenging to collect a sufficient number of cases. Specifically, the inclusion of these JIA patients potentially confounded the analysis of both pain and medication parameters. Finally, our findings lend credence to the necessity of conducting additional research to further explain the nature and etiology of MSK symptoms, as well as their impact on HRQoL, and to identify the most effective treatment techniques for patients who are experiencing these symptoms.

## Conclusion

In conclusion, MSK manifestations are common and diverse in CNO patients and are more common in females and in those with associated psoriasis. MSK manifestations have a negative impact on HRQoL in CNO patients, and thus, early identification and treatment are highly recommended.

### Electronic supplementary material

Below is the link to the electronic supplementary material.

Supplementary Material 1

## Data Availability

The datasets used and/or analysed during the current study are available from the corresponding author on reasonable request.

## Abbreviations

bDMARDs: Biological disease-modifying antirheumatic drugs
cDMARDs: Conventional disease-modifying antirheumatic drugs
CNO: Chronic nonbacterial osteomyelitis
HRQoL: Health-related quality of life
IBD: Inflammatory bowel disease
MSK: Musculoskeletal
MTX: Methotrexate
NMQ-E: Nordic Musculoskeletal Questionnaire
NSAIDs: Nonsteroidal anti-inflammatory drugs
PedsQL-4: Pediatric Quality of Life Inventory-4
SSZ: Sulfasalazine
TNF-α: Tumor necrosis factor alpha
