# Supplementary material for: Musculoskeletal symptoms and their impact on health-related quality of life in chronic nonbacterial osteomyelitis patients
Source: Pediatr Rheumatol Online J. 2024 Mar 6;22:34. doi: 10.1186/s12969-024-00971-7 (PMC10916259; doi:10.1186/s12969-024-00971-7)
Supplement: Supplementary file 1 — Supplementary Material 1 [file 12969_2024_971_MOESM1_ESM.docx]

Cover letter

Dated 28/12/2023.

Dear editor,

I have the pleasure of sending you the manuscript entitled “Musculoskeletal symptoms and their impact on health-related quality of life in chronic nonbacterial osteomyelitis patients” authored by Samar Tharwat^1,2^, Mohammed Kamal Nassar^3^ to be considered for publication as a research article in your prestigious journal “Pediatric Rheumatology”. Paper is containing original research and has not been submitted / published earlier in any journal and is not being considered for publication elsewhere. All authors have seen and approved the manuscript and have contributed significantly for the paper.

Ethical procedure

- The research meets all applicable standards regarding the ethics of experimentation and research integrity, and the following is being certified/declared true.
- As an expert scientist and along with co-authors of concerned field, the paper has been submitted with full responsibility, following due ethical procedure, and there is no duplicate publication, fraud, plagiarism, or concerns about animal or human experimentation.

A Disclosure / Conflict of Interest Statement

- All authors have contributed to the paper and have never submitted the manuscript, in whole or in part, to other journals.
- None of the authors of this paper has a financial or personal relationship with other people or organizations that could inappropriately influence or bias the content of the paper.
- It is to specifically state that “No Competing interests are at stake and there is No Conflict of Interest” with other people or organizations that could inappropriately influence or bias the content of the paper.

**Corresponding author** Samar Tharwat

**E-mail address** samartharwat2000@mans.edu.eg

**Fields of interest** Rheumatology, Immunology, Musculoskeletal ultrasound

**Address** Mansoura University Hospital, El Gomhouria St, Mansoura, Dakahlia Governorate

**Telephone** 01091784143

**Fax numbers** +2 050 2202834
